# Additions to the family *Junewangiaceae* (*Sordariomycetes*): novel species and new records from freshwater habitats in Southwestern China

**DOI:** 10.3389/fmicb.2025.1566263

**Published:** 2025-04-22

**Authors:** Wen-Peng Wang, Chuan-Gen Lin, Ting-Xiang Liu, Hong-Wei Shen, Zong-Long Luo

**Affiliations:** ^1^College of Agriculture and Biological Science, Dali University, Dali, Yunnan, China; ^2^School of Life Sciences, Guizhou Normal University, Guiyang, China; ^3^Co-Innovation Center for Cangshan Mountain and Erhai Lake Integrated Protection and Green Development of Yunnan Province, Dali University, Dali, Yunnan, China

**Keywords:** two new taxa, aquatic fungi, morphology, phylogeny, taxonomy

## Abstract

*Junewangiaceae* (*Sordariomycetes*) is a family with a relatively recent taxonomic history and a small number of described species. However, a major challenge within this family is the inability to distinguish between various genera based solely on the phylogenetic analysis. In this study, we introduced two new species, *Junewangia guangxiensis* and *J. synnematica*, which formed independent clades in phylogenetic analysis and displayed characteristics that were easily distinguishable from other species within this family. Additionally, three previously known species, *viz.*, *Dictyosporella aquatica*, *D. thailandensis*, and *J. thailandensis*, are reported from China for the first time. Furthermore, *D. bambusicola* is documented from a freshwater habitat. The results enhance our understanding of *Junewangiaceae* and provide some suggestions for addressing the taxonomic problems of this family in the future.

## Introduction

Biodiversity is one of the most significant topics globally in current times (Díaz, 2022). However, our current understanding of biodiversity remains limited, especially in the fungal realm (Wanasinghe et al., 2023). In freshwater ecosystems, many fungi form an integral part of the material cycle and energy flow, making them an integral part of freshwater ecosystems (Hyde et al., 2016; Grossart et al., 2019). Over the past decade, more than 200 new species of *Ascomycota*, including members of the family *Junewangiaceae*, have been discovered in freshwater habitats in China (Luo et al., 2019; Bao et al., 2021; Dong et al., 2020; Calabon et al., 2022; Shen et al., 2022; Yang et al., 2023). However, these discoveries still remain insufficient compared to the forecasted number of species (Bánki et al., 2023; Calabon et al., 2023). Therefore, relevant research should continue to be conducted on a broader scale in the future.

Ariyawansa et al. (2015) introduced *Dictyosporella* within the family *Annulatascaceae*. The asexual morph of *Dictyosporella* is characterized by reduced conidiophores, monoblastic, terminal, thin-walled conidiogenous cells with or without separating cells, and muriform, broadly ellipsoidal to subglobose conidia (Yuan et al., 2020; Dong et al., 2021; Hyde et al., 2023). Later, the phylogenetic analysis by Xia et al. (2017) established the *Junewangiaceae* family to accommodate the acrodictys-like genus *Junewangia*. However, subsequent phylogenetic analysis by Luo et al. (2019) showed that *Dictyosporella*, *Junewangia*, and *Sporidesmiella* clustered within *Junewangiaceae*, a conclusion that is supported by follow-up studies (Dong et al., 2021; Li et al., 2021; Hyde et al., 2023). Additionally, Goh and Kuo (2020) introduced *Jennwenomyces*, which was segregated from *Belemnospora*. Although phylogenetic analysis positioned *Jennwenomyces* within *Junewangiaceae*, Goh and Kuo (2020) classified it as a *Sordariomycetes* genus *insertae sedis*.

**Figure 1:**
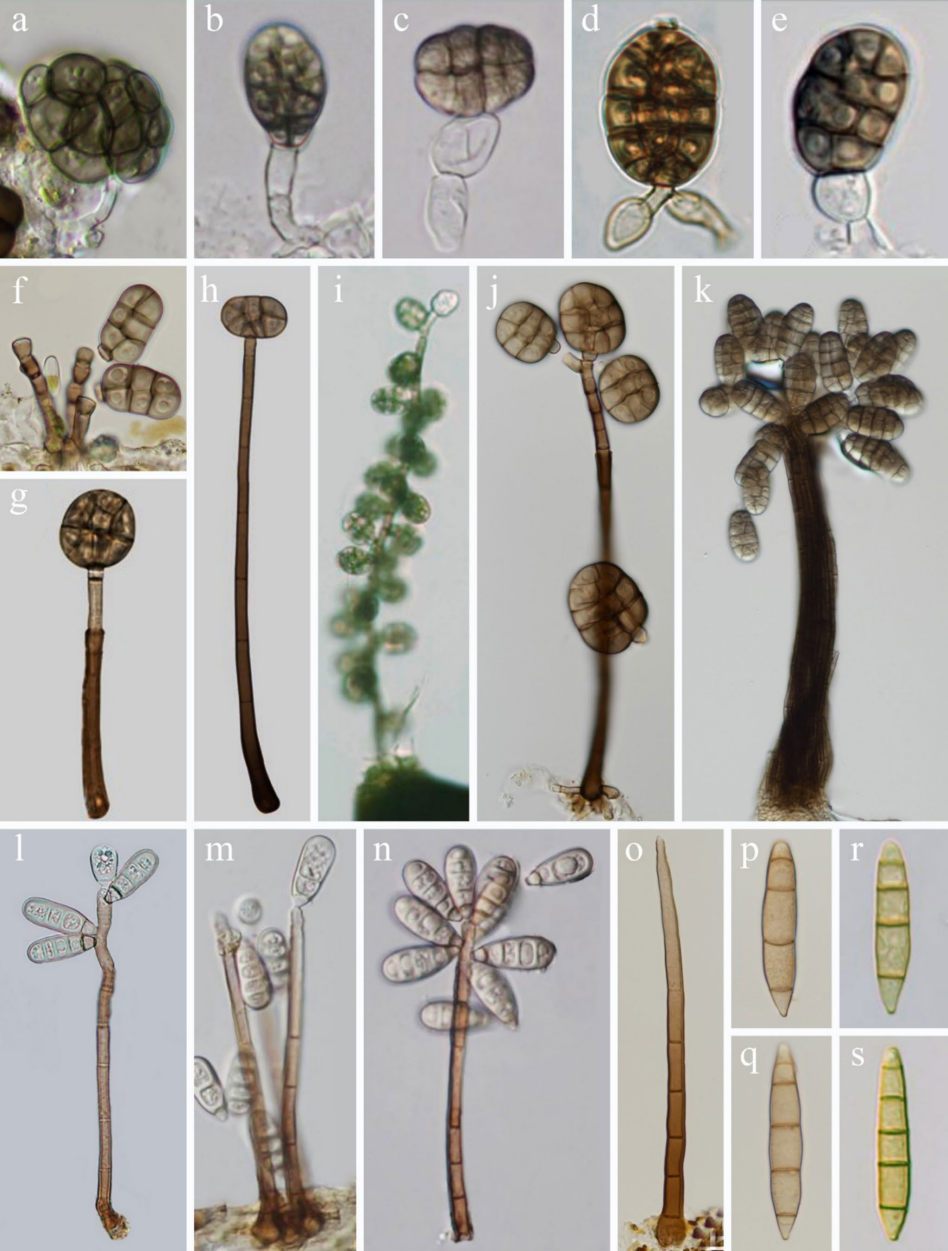
Asexual morph of *Junewangiaceae*. **(a–e)** *Dictyosporella* spp.; **(f–k)** *Junewangia* spp.; **(l–n)** *Sporidesmiella* spp.; **(o–s)** *Jennwenomyces fusiformis*.

Currently, the phylogenetic boundaries between *Dictyosporella*, *Junewangia*, *Jennwenomyces*, and *Sporidesmiella* remain unclear (Goh and Kuo, 2020; Hyde et al., 2023). These genera exhibit distinct morphological differences. Both *Dictyosporella* and *Junewangia* have muriform, broadly ellipsoidal to subglobose conidia, but *Junewangia* is differentiated by macronematous, mononematous, erect conidiophores with percurrent proliferations (Figure 1; Ariyawansa et al., 2015; Xia et al., 2017; Song et al., 2018a; Yuan et al., 2020; Dong et al., 2021; Hyde et al., 2023). *Jennwenomyces* resembles *Sporidesmiella* in having macronematous, mononematous, cylindrical, thick-walled conidiophores (Goh and Kuo, 2020). However, *Jennwenomyces* differs in having navicular, euseptate conidia, whereas *Sporidesmiella* produces obovoid or clavate, distoseptate conidia (Figure 1; Luo et al., 2019; Goh and Kuo, 2020; Li et al., 2021; Liu et al., 2024; Tian et al., 2024).

## Materials and methods

### Samples collection

Specimens of submerged decaying wood were collected from freshwater habitats (stream and river) in Yunnan Province (July 2023 and February 2024) and Guangxi Zhuang Autonomous Region (February 2024), China. The specimens were brought to the laboratory in plastic bags to preserve their integrity. The sample processing followed the method described by Shen et al. (2023): the samples were cut to the appropriate length, numbered, and placed in a disinfected plastic crisper for incubated culture at room temperature.

### Isolation and morphological examination

Fungal colonies on natural substrates were observed using a Guiguang GL-99BI compound stereomicroscope (Guilin Guiguang Instrument Co., Ltd., Guilin, China) and photographed with a Nikon SMZ1000 stereo zoom microscope (Nikon Corporation, Tokyo, Japan). Fungal structures were photographed using a Nikon ECLIPSE Ni-U compound microscope (Nikon Corporation, Tokyo, Japan) fitted with a Nikon DS-Ri2 digital camera (Nikon Corporation, Tokyo, Japan), according to the guidelines provided in the study by Luo et al. (2018). Fungal species were isolated using single spore isolation following the method outlined in the study by Senanayake et al. (2020). Germinating ascospores and conidia were transferred to fresh potato dextrose agar (PDA) media and incubated at room temperature. Herbarium specimens (dry woody branches with fungal material) were deposited in the Herbarium of Cryptogams at the Kunming Institute of Botany, Academia Sinica (HKAS), Kunming, China. The isolates obtained in this study were deposited in the China General Microbiological Culture Collection Center (CGMCC), Beijing, China and the Kunming Institute of Botany Culture Collection Center (KUNCC), Kunming, China. Names of the new taxa were registered in Fungal Names (FN).^1^ We followed the suggestions provided in the study by Thines et al. (2020) and italicized all the Latin names that appeared in the text.

### DNA extraction, PCR amplification, and sequencing

The genomic DNA was extracted from fungal mycelium. A Trelief™ Hi-Pure Plant Genomic DNA Kit (Beijing TsingKe Biotech Co., Ltd., Beijing, China) was used to extract total genomic DNA following the manufacturer’s instructions. DNA amplification was performed by polymerase chain reaction (PCR). the large subunit of nuclear ribosomal RNA gene (LSU), the nuclear ribosomal internal ranscribed spacer (ITS), the small subunit of nuclear ribosomal RNA gene (SSU), the translation elongation factor 1-alpha (*tef*1-*α*), and the second-largest subunit of RNA polymerase II (*rpb*2) gene regions were amplified using the primer pairs LR0R/LR5 (Vilgalys and Hopple, 1990), ITS5/ITS4 (White, 1990), NS1/NS4 (White, 1990), 983F/2218R (Rehner and Buckley, 2005), and fRPB2-5F/fRPB2-7cR (Liu et al., 1999). The amplifications were performed in a 25 μL reaction volume containing 9.5 μL ddH_2_O, 12.5 μL 2 × Taq PCR Master Mix with blue dye (Shanghai Sangon Biological Engineering Technology and Services Co., Shanghai, China), 1 μL DNA template, and 1 μL of each primer (10 μM). PCR products were checked on 1% agarose electrophoresis gels stained with GelRed (Beijing TsingKe Biotech Co., Ltd., Beijing, China). The sequencing reactions were carried out using the primers mentioned above by Shanghai Sangon Biological Engineering Technology and Services Co., Shanghai, China.

### Phylogenetic analyses

The Basic Local Alignment Search Tool (BLAST) searches in the National Center of Biotechnology Information (NCBI) preliminarily screened out strains of *Junewangiaceae*. Five gene markers, LSU, ITS, SSU, *tef*1-α, and *rpb*2, were used for the multigene analyses, with the whole or part of them concatenated for different fungal groups. Single-locus sequences were aligned using the online multiple alignment program MAFFT version 7 (Rozewicki et al., 2019), and this alignment was manually optimized in BioEdit version 7.0.5.3 (Hall, 1999). The concatenated sequence alignments were obtained from SequenceMatrix version 1.7.8 (Vaidya et al., 2011).

Maximum likelihood (ML) analysis was performed using Randomized Axelerated Maximum Likelihood High-Performance Computing 2 (RAxML-HPC2) on ACCESS (Stamatakis, 2006; Stamatakis et al., 2008) on the Extreme Science and Engineering Discovery Environment (XSEDE) TeraGrid of the CIPRES Science Gateway online platform (Miller et al., 2010) with rapid bootstrap analysis, which was followed by 1,000 bootstrap replicates. The final tree was selected among the suboptimal trees from each run by comparing the likelihood scores under the general time-reversible gamma (GTRGAMMA) parameter substitution model.

Bayesian inference (BI) analysis was performed in a likelihood framework implemented in MrBayes version 3.1.2 (Ronquist et al., 2012). The Markov Chain Monte Carlo (MCMC) sampling approach was used to calculate posterior probabilities (PP) (Rannala and Yang, 1996). A Bayesian analysis of six simultaneous Markov chains was run for 10,000,000 generations, with trees sampled at intervals of every 1,000 generations. The sequences generated in this study have been deposited in GenBank and are listed in Table 1.

**Table 1:** Strains/specimens used for phylogenetic analysis along with their GenBank accession numbers.

Species	Source	GenBank accession number				
		LSU	ITS	SSU	*tef*1-α	*rpb*2
*Acrodictys bambusicola*	CGMCC 3.18641	KX033564	KU999973	KX033535	_	_
*Acrodictys elaeidicola*	CGMCC 3.18642	KX033568	KU999977	KX033539	_	_
*Acrodictys elaeidicola*	CGMCC 3.18643	KX033569	KU999978	KX033540	_	_
*Acrodictys globulosa*	CGMCC 3.18644	KX033562	KU999970	KX033532	_	_
***Acrodictys hainanensis***	**CGMCC 3.18645**	**KX033565**	**KU999974**	**KX033536**	_	_
*Acrodictys peruamazonensis*	CGMCC 3.18649	KX033561	KU999969	KX033531	_	_
*Annulusmagnus triseptatus*	CBS 128831	GQ996540	_	JQ429242	_	JQ429258
*Annulatascus tratensis*	MFLUCC 17-2123	OP377972	OP377886	_	_	_
***Annulatascus tratensis***	**MFLUCC 17-2055**	**OP377977**	**OP377891**	**OP378052**	_	_
***Dictyosporella aquatica***	**CBS H-22127**	**KT241022**	_	**KT241023**	_	_
*Dictyosporella aquatica*	KUNCC 24-17687	PQ532938	_	_	_	_
*Dictyosporella aquatica*	KUNCC 24-17689	PQ532939	_	_	_	_
***Dictyosporella bambusicola***	**CGMCC 3.27442**	**PQ067725**	**PQ067809**	_	**PQ278561**	**_**
*Dictyosporella bambusicola*	KUNCC 23-16530	PQ532936	PQ532945	_	PQ665297	_
***Dictyosporella chiangmaiensis***	**MFLUCC 17-2345**	**MW287765**	**MW286491**	_	_	_
***Dictyosporella ellipsoidea***	**MFLUCC 18-1042**	**MW287758**	_	_	_	_
***Dictyosporella guizhouensis***	**MFLU 18-1505**	**MK593605**	**MK593606**	**MK593611**	_	_
*Dictyosporella guizhouensis*	MFLUCC 18-1232	MW287760	MW286487	_	MW396646	_
***Dictyosporella hydei***	**IFRDCC 3075**	**MG813161**	_	_	_	_
***Dictyosporella thailandensis***	**MFLUCC 15-0985**	**MF374364**	**MF374355**	**MF374373**	**MF370958**	**MF370952**
*Dictyosporella thailandensis*	KUNCC 24-18231	PQ532937	_	_	_	_
***Dictyosporella yunnanensis***	**ZHKUCC 22-0294**	**OP755246**	**OP755247**	_	_	_
***Distoseptispora euseptata***	**MFLUCC 20-0154**	**MW081544**	**MW081539**	_	_	**MW151860**
*Distoseptispora euseptata*	DLUCC S2024	MW081545	MW081540	_	MW084994	MW084996
***Distoseptispora saprophytica***	**MFLUCC 18-1238**	**MW287780**	**MW286506**	_	**MW396651**	**MW504069**
***Distoseptispora tectonae***	**MFLUCC 12-0291**	**KX751713**	**KX751711**	_	**KX751710**	**KX751708**
*Distoseptispora tectonae*	MFLUCC 15**-**0981	MW287763	MW286489	_	MW396641	_
*Jennwenomyces navicularis*	NCYU-JW1	MT224910	MT224911	_	_	_
*Jennwenomyces navicularis*	BCRC FU30872	MT224909	MT224914	_	_	_
*Jennwenomyces navicularis*	CGMCC 3.28462	PQ532935	PQ532943	_	_	_
*Jennwenomyces navicularis*	KUNCC 24-18625	PQ655534	PQ655536	_	_	_
*Jennwenomyces navicularis*	KUNCC 24-18632	PQ655535	PQ655537	_	_	_
*Jennwenomyces* sp.	KUNCC 24-18125	PQ152623	PQ168237	_	_	_
***Junewangia aquatica***	**HFJAU 0700**	**MG213737**	**MG213738**	**MG213736**	_	_
***Junewangia guangxiensis***	**CGMCC 3.28461**	**PQ532940**	**PQ532946**	**PQ555249**	**PQ665296**	_
*Junewangia lamma*	HMAS 44438	KU751882	KU999961	KX033523	_	_
*Junewangia lamma*	HSAUP H4695	KU751883	KU999971	KX033533	_	_
*Junewangia lamma*	MFLUCC 23-0258	OR826594	OR826589	OR826600	_	_
*Junewangia. queenslandica*	CGMCC 3.18654	KX033575	KU999984	KX033546	_	_
*Junewangia sphaerospora*	CGMCC 3.18655	KX033572	KU999981	KX033543	_	_
***Junewangia synnematica***	**KUNCC 23-16608**	**PQ532941**	**PQ532947**	**PQ555250**	**PQ671443**	**PQ671445**
*Junewangia synnematica*	KUNCC 24-19062	PQ532942	PQ532948	PQ555251	PQ671444	PQ671446
***Junewangia thailandica***	**MFLU 15-2682**	**MW287762**	_	_	_	_
*Junewangia thailandica*	KUNCC 24-18384	PQ532943	_	_	_	_
***Sporidesmiella aquatica***	**DLUCC 0777**	**MK849843**	**MK828692**	_	**MN194034**	_
*Sporidesmiella aquatica*	MFLU 18-1602	_	NR 168811	_	_	_
*Sporidesmiella hyalosperma*	MFLUCC 18-1013	MW287773	MW286499	_	MW396654	MW504070
*Sporidesmiella hyalosperma*	MFLUCC 18-1312	MK849839	MK828688	_	MN194031	MN124520
*Sporidesmiella novae-zelandiae*	S-1256	MK849845	MK828693	_	MN194036	MN124525
*Sporidesmiella novae-zelandiae*	S-951	MK849847	MK828695	_	MN194037	MN124526
***Sporidesmiella obovoidia***	**MFLUCC 17-2372**	**NG 075412**	**NR 172446**	_	_	_
*Thyridium vestitum*	AFTOL-ID 172	AY544671	_	AY544715	DQ471058	DQ470890

The ex-type species, strains, and sequences are presented in bold, while the newly generated ones are indicated in red.

## Results

### Phylogenetic analyses

The dataset that includes combined LSU, ITS, SSU, *tef*1-*α*, and *rpb*2 sequence data comprises 52 strains with 4,092 characters, including gaps (LSU: 1–802 bp, ITS: 803–1,310 bp, SSU: 1,311–2,178 bp, *tef*1-α: 2,179–3,042 bp, and *rpb*2: 3,043–4,092 bp). *Thyridium vestitum* (AFTOL-ID 172) was selected as the outgroup taxon. RAxML and Bayesian analyses were conducted and resulted in generally congruent topologies. The best RAxML tree with a final likelihood value of −21,682.843109 is presented. The matrix contained 1,439 distinct alignment patterns, with 53.62% undetermined characters or gaps. The estimated base frequencies were as follows: A = 0.241236, C = 0.253535, G = 0.284886, T = 0.220342; the substitution rates were as follows: AC = 1.104233, AG = 2.702435, AT = 1.264711, CG = 0.798496, CT = 6.288150, and GT = 1.000000; the gamma distribution shape parameter was *α* = 0.205348.

**Figure 2:**
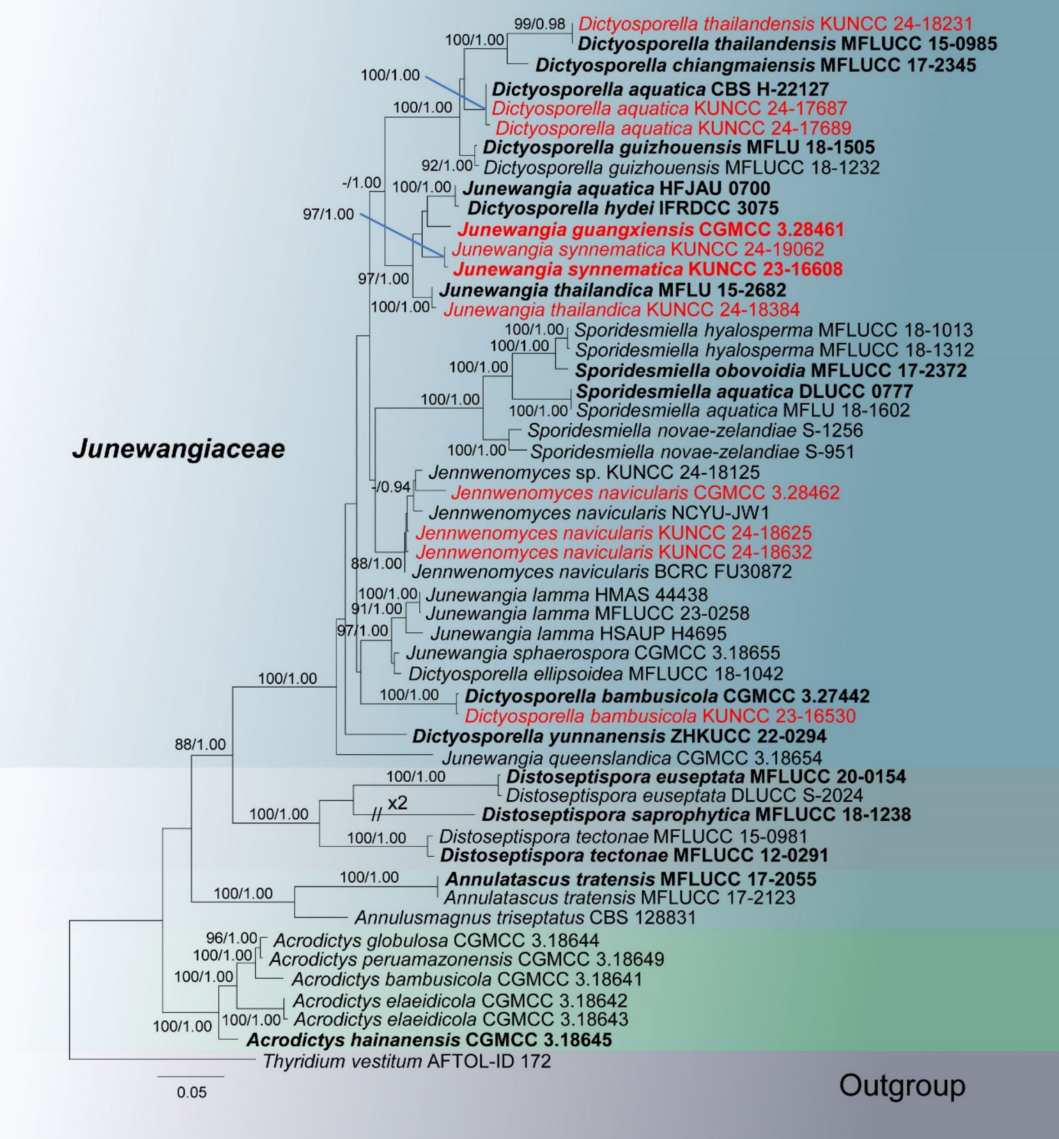
RAxML tree based on combined LSU, ITS, SSU, *tef*1-*α*, and *rpb*2 sequence date of *Junewangiaceae*. Bootstrap support values for maximum likelihood (ML) greater than 80% and Bayesian posterior probabilities (PP) greater than 0.90 are given as ML/PP above the nodes. The newly obtained sequences are indicated in red and ex-type strains are indicated in bold.

In the phylogenetic tree, 11 newly obtained strains are nested within the family *Junewangiaceae* (Figure 2). The species *Junewangia guangxiensis* (CGMCC 3.28461) and *J. synnematica* (KUNCC 23-16608 and KUNCC 24-19062) cluster together with *Dictyosporella hydei* (IFRDCC 3075), *J. aquatica* (HFJAU 0700), and *J. thailandica* (MFLU 15-2682 and KUNCC 24-18384) in the same clade with 97% ML/1.00 PP support. Three new collections of *Jennwenomyces navicularis* (CGMCC 3.28462, KUNCC 24-18625, and KUNCC 24-18632) grouped with the clade of *Je. navicularis* with 88% ML/1.00 PP support. Other four known species *D. aquatica* (KUNCC 24-17687 and KUNCC 24-17689), *D. bambusicola* (KUNCC 23-16530), *D. thailandensis* (KUNCC 24-18231), and *J. thailandica* (KUNCC 24-18384) cluster with their holotype or ex-type strains with 100% ML/1.00 PP, 100% ML/1.00 PP, 99% ML/0.98 PP, and 100% ML/1.00 PP support, respectively (Figure 2).

### Taxonomy

***Dictyosporella aquatica*** Abdel-Aziz, Fungal Diversity 75: 119 (2015), Figure 3.

**Figure 3:**
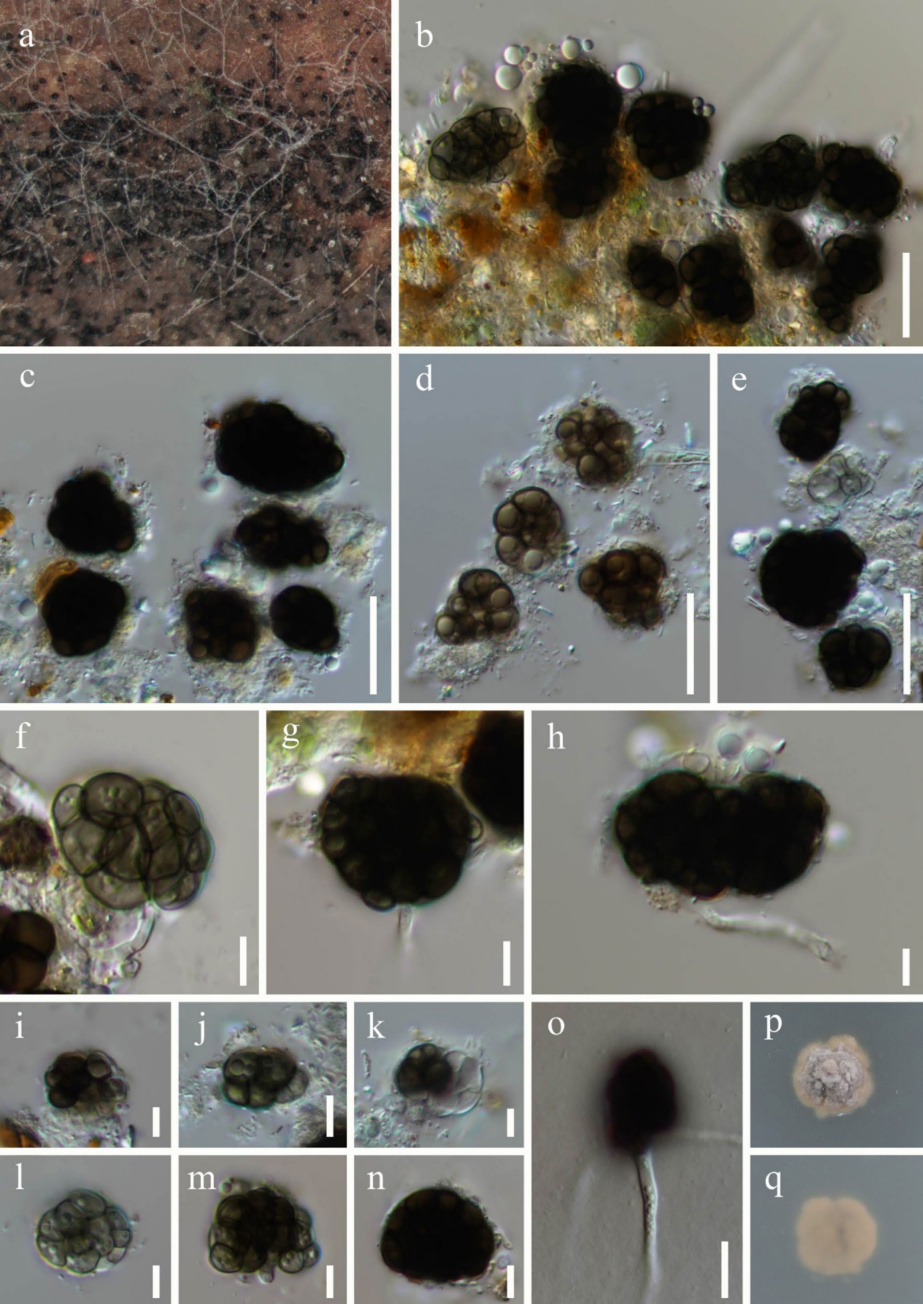
*Dictyosporella aquatica* (HKAS 144562). **(a)** Colonies on the substratum; **(b–e,i–n)** Conidia; **(f–h)** Conidiophores with conidia; **(o)** Germinating conidium; **(p)** Colony on PDA on the surface; **(q)** Colony on PDA on reverse. Scale bars: **(b–e)** 20 μm; **(f–n)** 5 μm; **(o)** 10 μm.

Fungal names number: FN551481.

*Saprobic* on submerged stems of *Bambusoideae* sp. **Asexual morph**: *Colonies* – superficial, effuse, gathered or scattered, dark brown to black, glistening. *Mycelium* – partly immersed, partly superficial, composed of aseptate, smooth, hyaline hyphae. *Conidiophores* – mostly reduced, mononematous, semi-macronematous, cylindrical, hyaline. *Conidiogenous cells* – monoblastic, integrated, terminal, hyaline. *Conidia* 16–29 × 9.8–24 μm ( $\bar{x}$ = 22.1 × 16 μm, *n* = 30), acrogenous, solitary, helicoid when young, later becoming irregularly shaped, composed of subglobose to globose or irregular cells, guttulate, olive or brown when immature, dark brown when mature. **Sexual morph**: Undetermined.

*Culture characteristics*: Conidia germinating on PDA within 36 h and germ tubes. Colonies on PDA reaching 10 mm diameter after 8 weeks at room temperature. Colonies on the surface of PDA, protruding, with regular edges, dry, surface rough, grey to pale brown; pale brown, smooth from reverse.

*Material examined:* China, Guangxi Zhuang Autonomous Region, Nanning City (22°49′48″N; 108°13′32″E), on submerged *Bambusoideae* sp. in a freshwater river, 17 November 2023, Qiu-Xia Yang, S-5975 (HKAS 144562), living culture, KUNCC 24-17687; *ibid.*, S-5977 (HKAS 144563), living culture, KUNCC 24-17689.

*Notes:* In the phylogenetic tree, two new collections (KUNCC 24-17687 and KUNCC 24-17689) were clustered with the ex-type strain of *Dictyosporella aquatica* (CBS H-22127) with 100% ML and 1.00 PP support (Figure 2). Our new collections resemble *D. aquatica* in having solitary conidia that are helicoid when young, later becoming irregular in shape, composed of subglobose to globose cells (Ariyawansa et al., 2015). We therefore identify our new collections as *D. aquatica*, a species introduced by Ariyawansa et al. (2015) on submerged decayed stems of *Phragmites australis* (*Poaceae*) in Egypt. This is the first report of this species in China.

***Dictyosporella bambusicola*** X.D. Yu & Jian K. Liu, Mycosphere 15(1): 5104 (2024), Figure 4.

**Figure 4:**
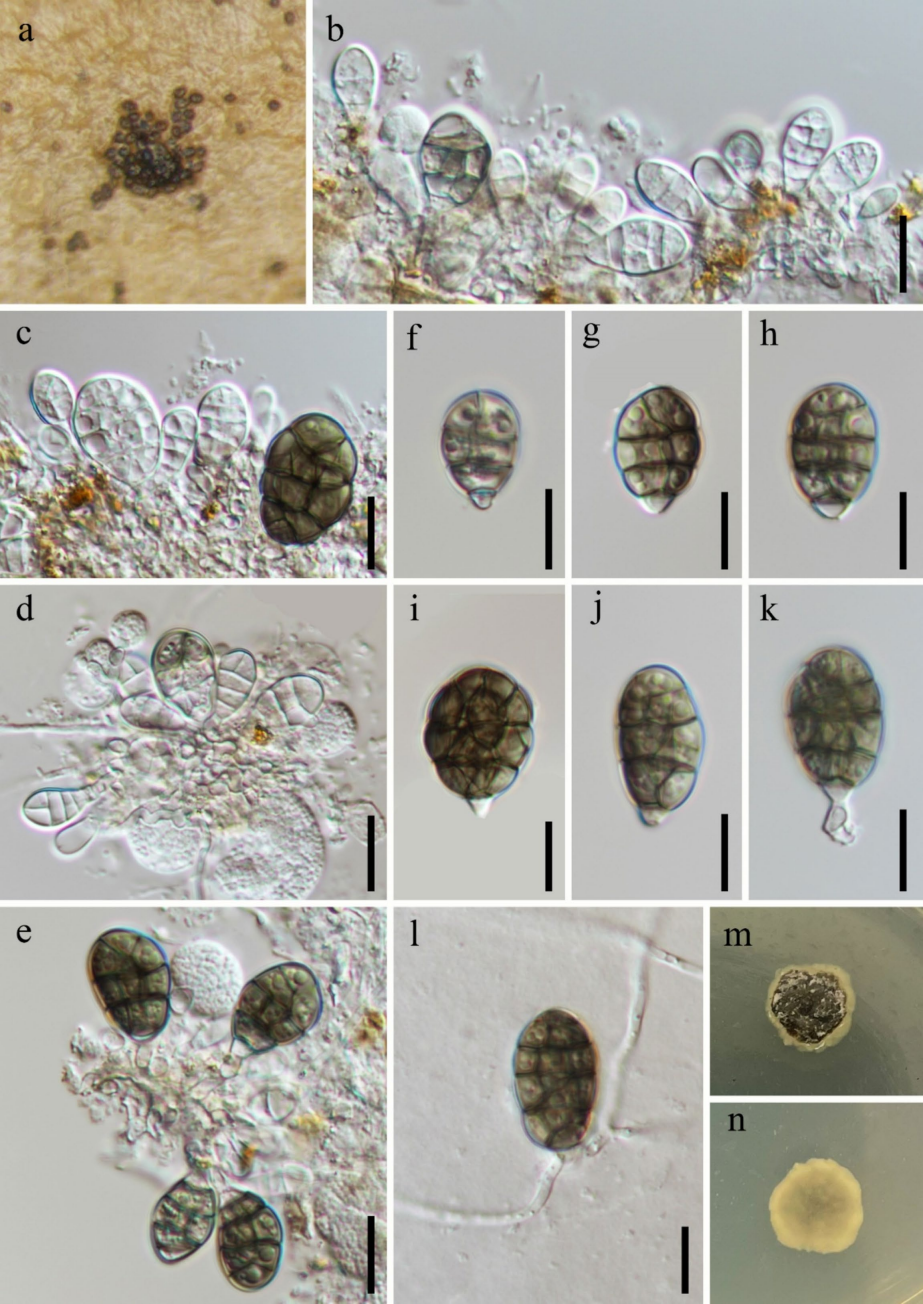
*Dictyosporella bambusicola* (HKAS 144564). **(a)** Colonies on the substratum; **(b–e)** Conidiophores with conidia; **(f–k)** Conidia; **(l)** Germinating conidium. **(m)** Colony on the surface of PDA; **(n)** Colony on the reverse of PDA. Scale bars: **(b–l)** 10 μm.

Fungal names number: FN854894.

*Saprobic* on submerged stems of *Bambusoideae* sp. **Asexual morph**: *Colonies* – superficial, effuse, sporodochia, brown. *Mycelium* – immersed, composed of aseptate, smooth, hyaline hyphae. *Conidiophores* – mostly reduced, semi-macronematous to micronematous, mononematous, unbranched, cylindrical, hyaline, aseptate, smooth and thin-walled. *Conidiogenous cells* 2.5–5.9 × 1.3–2.5 μm ( $\bar{x}$ = 3.3 × 1.8 μm, *n* = 10), monoblastic, integrated, determinate, terminal, cylindrical, hyaline. *Conidia* 12–23 × 9.3–18 μm ( $\bar{x}$ = 18 × 12.7 μm, *n* = 40), acrogenous, solitary, ellipsoidal, muriform, complanate, longitudinal or oblique and transverse separation, slightly constricted at the septum, composed of 3–4 rows of cells with a paler basal cell, hyaline when immature and becoming brown when mature, guttulate. **Sexual morph**: Undetermined.

*Culture characteristics*: Conidia germinating on PDA within 24 h and germ tubes produced from the base. Colonies on PDA reaching 8 mm diameter after 4 weeks at room temperature. Colonies on the surface of PDA, protruding, dry, with regular edges, surface rough, dark brown with a light brownish-yellow, gelatinous edge, light brownish-yellow, smooth from reverse.

*Material examined:* China, Yunnan Province, Honghe Hani and Yi Autonomous Prefecture, Mile City (24°42′69.75″N; 103°48′34.68″E), on submerged *Bambusoideae* sp. in a freshwater stream, 14 July 2023, Xing-Ya Zeng, S-5433 (HKAS 144564), living culture, KUNCC 23-16530.

*Notes:* Phylogenetic analysis revealed that our new collection clustered with the ex-type strain of *Dictyosporella bambusicola* with 100% ML and 1.00 PP support (Figure 2). Morphologically, our new collection exhibits micronematous, cylindrical conidiophores and ellipsoidal, muriform conidia, which are similar to *D. bambusicola* (Yu et al., 2024). A comparison of the ITS, LSU, and *tef*1-*α* sequence similarity between the new collection and *D. bambusicola* showed that 99.77% (434/435 bp), 100% (814/814 bp), and 99.45% (905/910 bp) similarity, respectively. We therefore identify our new collection as *D. bambusicola* based on phylogeny and morphological characteristics.

***Dictyosporella thailandensis*** W. Dong, H. Zhang & K.D. Hyde, Fungal Diversity 85: 33 (2017), Figure 5.

**Figure 5:**
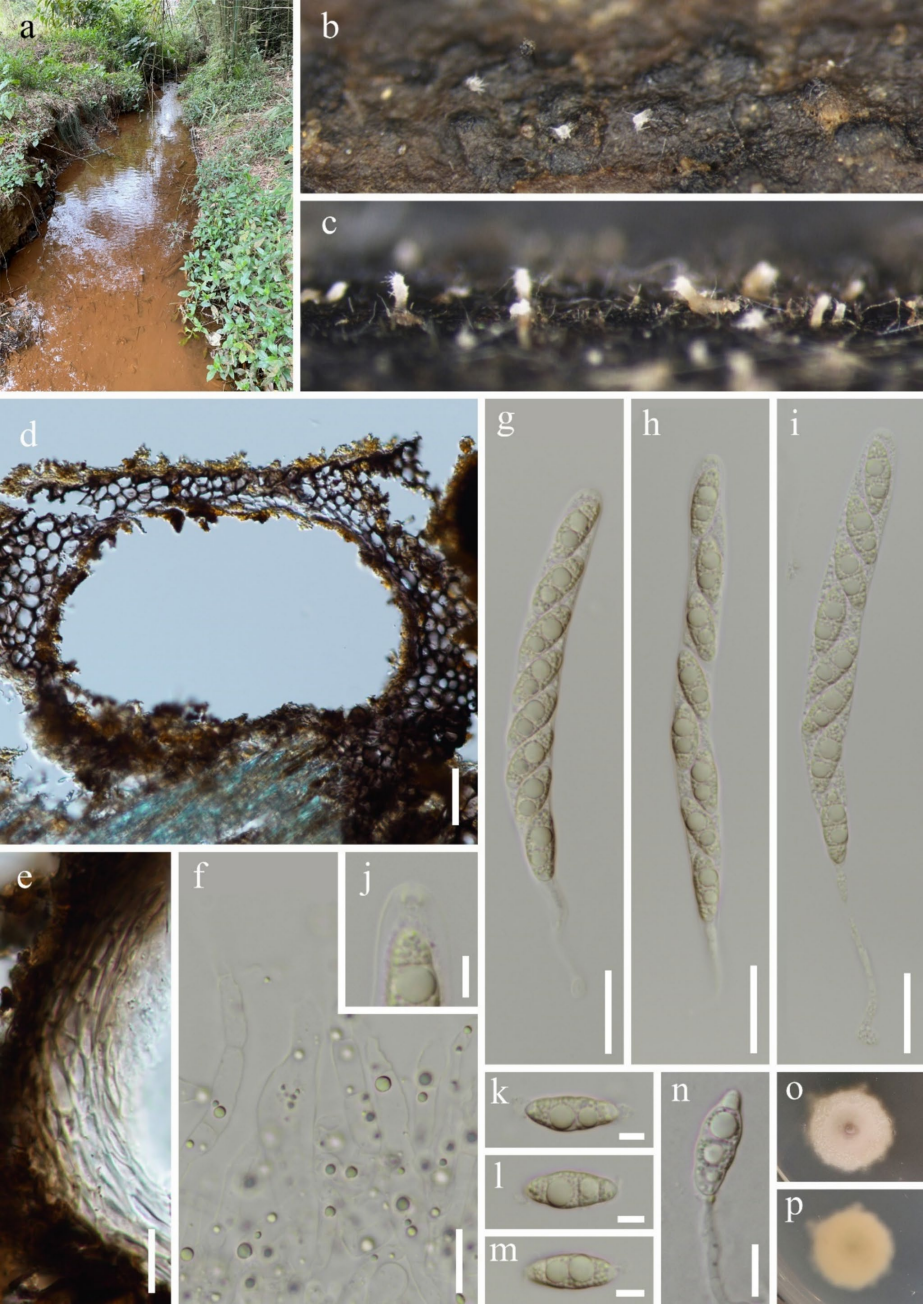
*Dictyosporella thailandensis* (HKAS 144552). **(a)** Freshwater habitat; **(b)** and **(c)** Ascomata on the substratum; **(d)** Vertical section of ascoma; **(e)** Structure of peridium; **(f)** Paraphyses; **(g–i)** Asci; **(j)** Apex of ascus; **(k–m)** Ascospores; **(n)** Germinating ascospore; **(o)** Colony on the surface of PDA; **(q)** Colony on the reverse of PDA. Scale bars: **(d)** 30 μm; **(e,f,n)** 10 μm; **(g–i)** 20 μm; **(j,k–m)** 5 μm.

Fungal names number: FN553770.

*Saprobic* on submerged decaying wood. **Asexual morph**: Undetermined. **Sexual morph**: *Ascomata* 102–200 μm height and 101–197 μm diameter, scattered, solitary or gregarious, semi-immersed, with neck erumpent through host surface, uniloculate, ellipsoidal to subglobose, dark brown to black. *Neck* – cylindrical, central or lateral, hyaline to pale brown, periphysate, with multiple fluff appendages. *Peridium* 5.9–33 μm thickness, coriaceous, two-layered, outer layer dark brown to black, inner layer comprising multiple rows of hyaline to pale brown, thick-walled, irregular cells *taxtura prismatica*. *Paraphyses* 2.6–6 (−10) μm wide, hyaline, septate, slightly constricted at the septum, unbranched, tapering toward the apex, guttulate. *Asci* 113–167 (−180) × 9–14 μm ( $\bar{x}$ = 142.4 × 11.5 μm, *n* = 20), eight-spored, unitunicate, cylindrical, with up to 43 μm long, tapering or dilated at the base pedicellate, rounded at the apex, with a refractive, wedge-shaped, J-, apical ring. *Ascospores* 15–19 × 5.6–7.3 μm ( $\bar{x}$ = 17.5 × 6.4 μm, *n* = 30), oblique uniseriate, ellipsoidal, straight, hyaline, tri-septate, slightly constricted at the septum, guttulate, with dull and filamentous appendages at both ends.

*Culture characteristics*: Ascospores germinating on PDA within 12 h and germ tubes produced from one end. Colonies on PDA reaching 10 mm diameter after 4 weeks at room temperature. Colonies on the surface of PDA, flat surface with a small protrusion in the center, irregular edges, dry, white; white to pale brown, smooth from reverse

*Material examined:* China, Guangxi Zhuang Autonomous Region, Wuzhou City (23°43′47.98″N; 110°85′96.75″E), on unknown submerged decaying wood in a freshwater stream, 23 February 2024, Fa-Li Li, S-6304 (HKAS 144552), living culture, KUNCC 24-18231.

*Notes: Dictyosporella thailandensis* was introduced by Zhang et al. (2017) from a freshwater habitat in Thailand. This species is characterized by subglobose or ellipsoidal ascomata with hyaline to pale yellow neck erumpent through the host surface, unitunicate, long cylindrical, pedicellate asci with an apical ring, and straight, 3-septate ascospores with filamentous bipolar appendages (Zhang et al., 2017). Our new collection matches the characteristics of *D. thailandensis*, and the phylogenetic analysis also supports identifying our new collection as *D. thailandensis*. We therefore identify our new collection as *D. thailandensis*, and this is the first report of this species in China.

***Jennwenomyces navicularis*** (R.F. Castañeda & Heredia) Goh &C.H. Kuo, Mycological Progress 19: 874 (2020), Figure 6.

**Figure 6:**
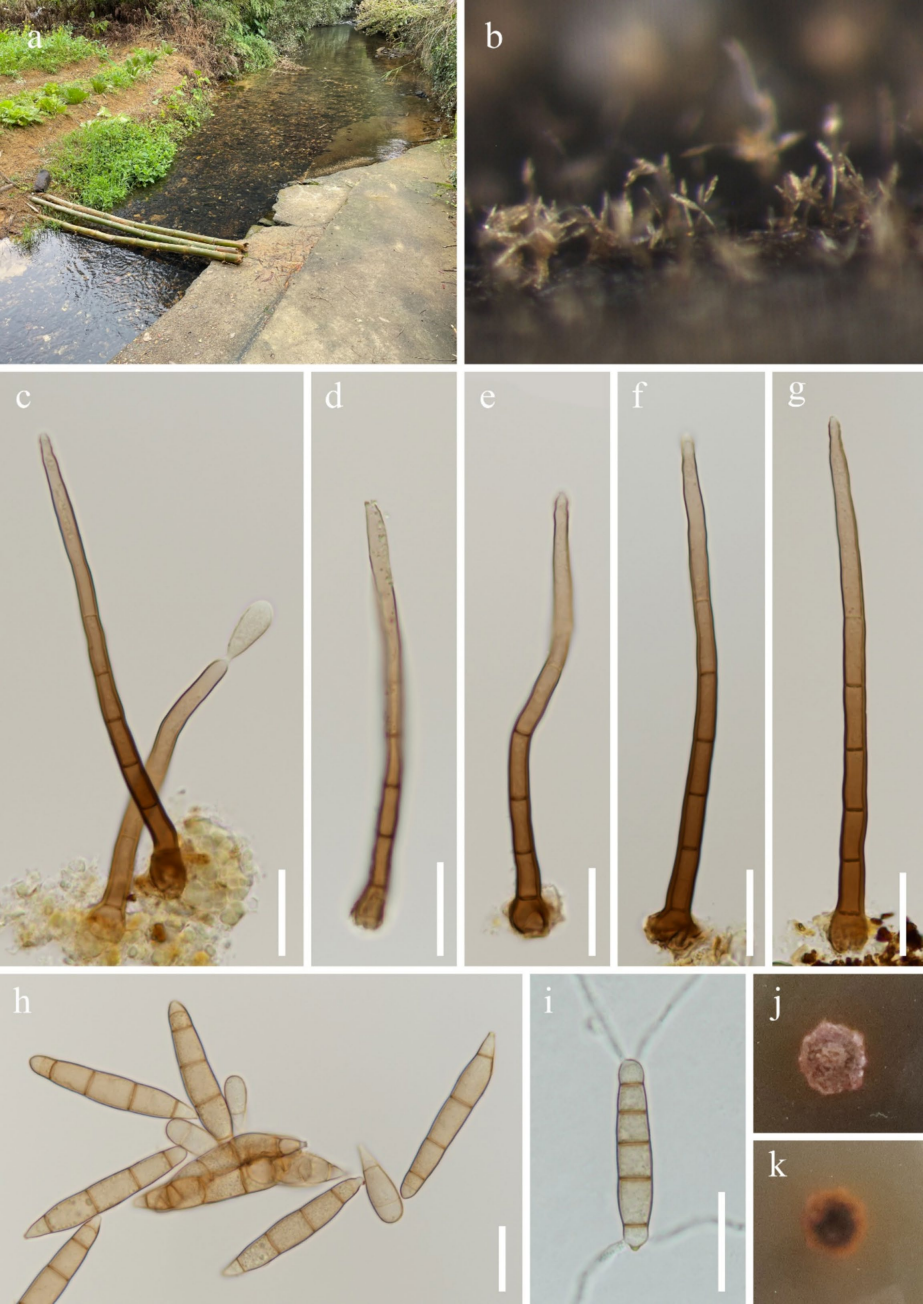
*Jennwenomyces navicularis* (HKAS 144559). **(a)** Freshwater habitat; **(b)** Colonies on the substratum; **(c–g)** Conidiophores with conidium; **(h)** Conidia; **(i)** Germinating conidium; **(j)** Colony on the surface of PDA; **(k)** Colony on the reverse of PDA. Scale bars: **(c–i)** 20 μm.

Fungal names number: FN835023.

*Saprobic* on submerged stems of *Bambusoideae* sp. **Asexual morph**: *Colonies* – superfical, scattered, effuse, brown. *Mycelium* – mostly immersed, composed of aseptate, smooth, hyaline hyphae. *Conidiophores* 66–133 × 3.2–6.1 μm ( $\bar{x}$ = 102.5 × 4.4 μm, *n* = 20), macronematous, mononematous, unbranched, cylindrical, 2–5-septate, straight or slightly flexuous, reddish-brown, paler toward the apex, smooth and thick-walled. *Conidiogenous cells* 36–56 × 3.3–5 μm ( $\bar{x}$ = 42.4 × 4 μm, *n* = 20), polyblastic, integrated, terminal, determinate, sympodial. *Conidia* 33–64 × 5.9–10 μm ( $\bar{x}$ = 49.6 × 8.6 μm, *n* = 20), acrogenous, solitary, smooth-walled, clavate when immature, becoming navicular when mature, tapering at both ends, with a rounded apex and a narrow hilum at the base, straight, 4(-5)-euseptate, partly with an inconspicuous septate at the apex cell, pale reddish-brown. **Sexual morph**: Undetermined.

*Culture characteristics*: Conidia germinating on PDA within 12 h and germ tubes produced from both ends. Colonies on PDA reaching 8 mm diameter after 6 weeks at room temperature. Colonies on the surface of PDA, surface rough, dry, regular edges, reddish-brown; dark brown in the center with reddish-brown edges from reverse.

*Material examined:* China, Guangxi Zhuang Autonomous Region, Guigang City (23°82′91.20″N; 110°26′68.92″E), on submerged *Bambusoideae* sp. in a freshwater stream, 23 February 2024, Tian-Tian Zhao, S-6457 (HKAS 144559), living culture, CGMCC 3.28462 = KUNCC 24-18389; Hechi City (24°55′70.58″N; 107°21′44.02″E), on unknown submerged decaying wood in a freshwater stream, 19 February 2024, Tian-Tian Zhao, S-6135 (HKAS 144551), living culture, KUNCC 24-18625; Baise City (24°08′90.39″N; 106°64′83.78″E), on unknown submerged decaying wood in a freshwater stream, 18 February 2024, Tian-Tian Zhao, S-6358 (HKAS 144553), living culture, KUNCC 24-18632.

*Notes:* Goh and Kuo (2020) established *Jennwenomyces* to accommodate *J. navicularis* which was transferred from *Belemnospora*. In the phylogenetic analysis, our new collections grouped with *J. navicularis* with 88% and 1.00 PP support (Figure 2). Comparison of the internal transcribed spacer (ITS) sequence of the new collection (CGMCC 3.28462) with *J. navicularis* (BCRC FU30872 and NCYU-JW1) and *Jennwenomyces* sp. (KUNCC 24-18125) revealed differences of 4.20% (22/524 bp, eight gaps), 5.15% (27/524 bp, eight gaps), and 4.58% (24/524 bp, eight gaps). However, our new collections have unbranched, cylindrical conidiophores and navicular, euseptate conidia with rounded apex, which fits well with the description of *J. navicularis*. Although there are significant differences between the ITS sequences of the new collection (CGMCC 3.28462) and *J. navicularis* (BCRC FU30872 and NCYU-JW1), we can identify our new collections as *J. navicularis* based on phylogenetic analysis and morphological characteristics. Moreover, studying this genus requires the discovery of more species in the future.

**Figure 7:**
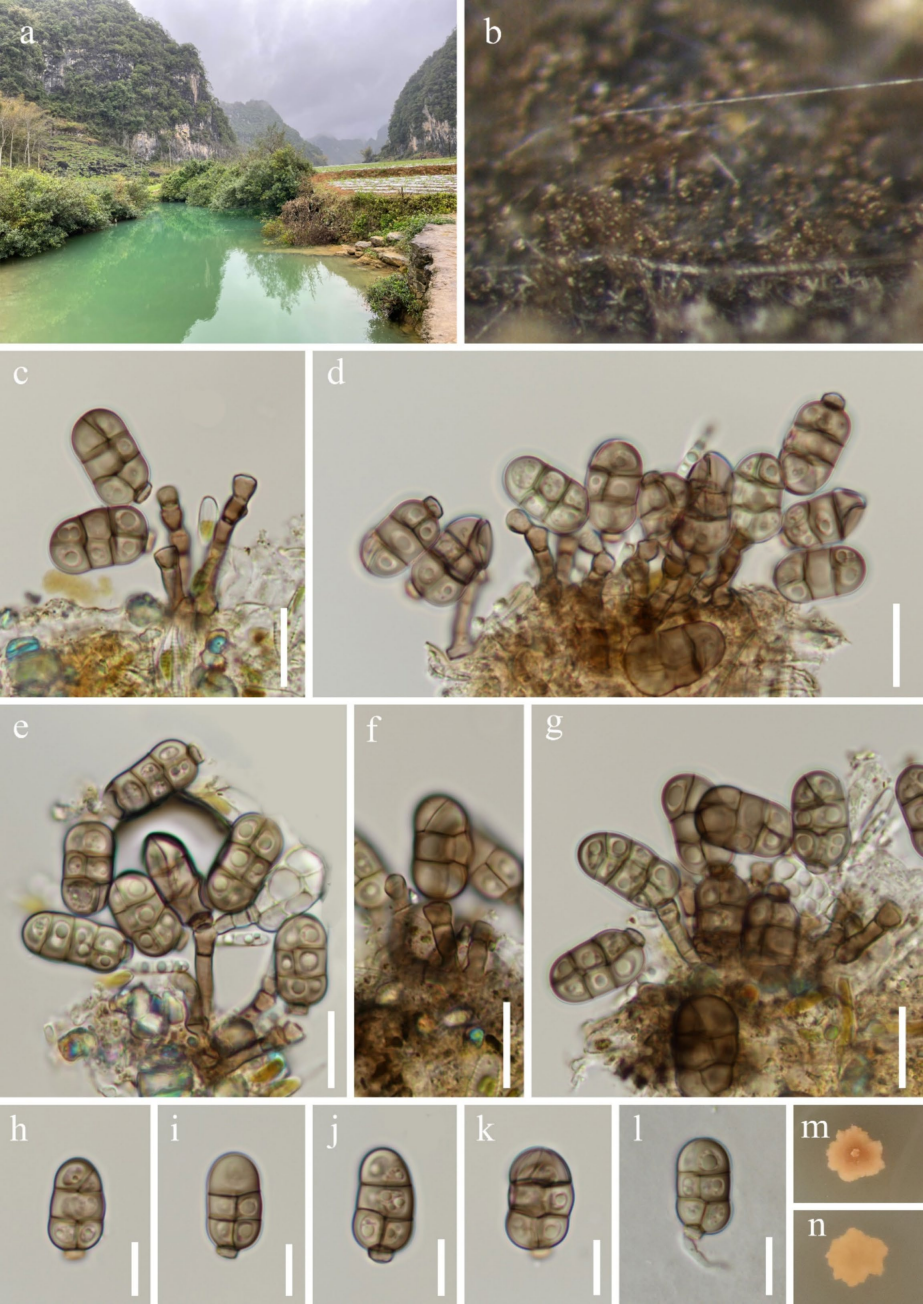
*Junewangia guangxiensis* (HKAS 144567, holotype). **(a)** Freshwater habitat; **(b)** Colonies on the substratum; **(c–g)** Conidiophores with conidia; **(h–k)** Conidia; **(l)** Germinating conidium; **(m)** Colony on the surface of PDA; **(n)** Colony on the reverse of PDA. Scale bars: **(c–g)** 15 μm; **(h–l)** 10 μm.

***Junewangia guangxiensis*** W.P. Wang & Z.L. Luo, sp. nov., Figure 7.

Fungal names number: FN572298.

Etymology: Referring to the Guangxi Zhuang Autonomous Region, China, where the species was collected.

Holotype: HKAS 144567.

*Saprobic* on submerged decaying wood. **Asexual morph**: *Colonies* – effuse, scattered, gathered in small groups, and brown. *Mycelium* – mostly immersed, composed of aseptate, smooth, and pale brown hyphae. *Conidiophores* 8.4–33 × 2.5–3.7 μm ( $\bar{x}$ = 18.1 × 3 μm, *n* = 30), macronematous, mononematous, erect, cylindrical, straight or slightly flexuous, 1–4-septate, unbranched, brown, smooth, thick-walled, with 0–1 percurrent proliferations. *Conidiogenous cells* – monoblastic, integrated, terminal, cuneiform to doliiform, pale brown. *Conidia* – 17–23 × 8.5–12 μm ( $\bar{x}$ = 20.1 × 10.5 μm, *n* = 40), acrogenous, solitary, ellipsoidal, muriform, composed of two columns cells with a cuneiform basal cell, longitudinal or oblique, and transverse separation, slightly constricted at the septum, partly rows without longitudinal septate, 1–2 cells at the apex row, rounded apical, brown, guttulate. **Sexual morph**: Undetermined.

*Culture characteristics*: Conidia germinating on PDA within 24 h and germ tubes produced from the base. Colonies on PDA reaching 8 mm diameter after 9 weeks at room temperature. Colonies on the surface of PDA, regular edges, dry, with a small, punctate protrusion in the center, reddish-brown to brown; brown and smooth from reverse.

*Material examined:* China, Guangxi Zhuang Autonomous Region, Nanning City (23°02′84.57″N; 107°52′41.73″E), on unknown submerged decaying wood in a freshwater stream, 28 February 2024, Zheng-Quan Zhang, S-6434 (HKAS 144567, holotype), ex-type culture, CGMCC 3.28461 = KUNCC 24-18376.

*Notes: Junewangia guangxiensis* has macronematous, erect, thick-walled conidiophores, which align well with the generic concept of *Junewangia* (Baker et al., 2002; Xia et al., 2017; Dong et al., 2021). *Junewangia guangxiensis* has short conidiophores that resemble *J. sphaerospora* (Table 2). However, *J. guangxiensis* differs from *J. sphaerospora* by having fewer percurrent proliferations (0–1 vs. 1–5 in *J. sphaerospora*), cuneiform to doliiform conidiogenous cells, and ellipsoidal, muriform, narrower conidia (8.5–12 vs. 12–18 μm wide) (Xia et al., 2017). Phylogenetic analysis also supports *J. guangxiensis* as a distinct species (Figure 2). We therefore recognize *J. guangxiensis* as a new species.

**Table 2:** Comparison of the conidiophores of different species of *Junewangia*.

Species	Morphology	Size (μm)	Septate	Percurrent extension
*Junewangia aquatica*	Mononematous	280–335 × 5–6	Multiple	Yes
*Junewangia guangxiensis*	Mononematous	8.4–33 × 2.5–3.7	1–4	Yes (0–1)
*Junewangia lamma*	Mononematous	8–55 × 2.5–3.5	1–5	Yes (1–3)
*Junewangia obliqua*	Mononematous	Up to 120 × 5–6	Multiple	Yes (1–2)
*Junewangia queenslandica*	Mononematous	103–142 × 3–4.5	5–8	Yes (0–2)
*Junewangia sphaerospora*	Mononematous	Up to 35 × 2.5–4	1–4	Yes (1–5)
*Junewangia synnematica*	Synnematous	102–137 × 2–2.3	Multiple	No
*Junewangia thailandica*	Mononematous	70–195 × 4–5.5	5–7	Yes (3–7)

**Figure 8:**
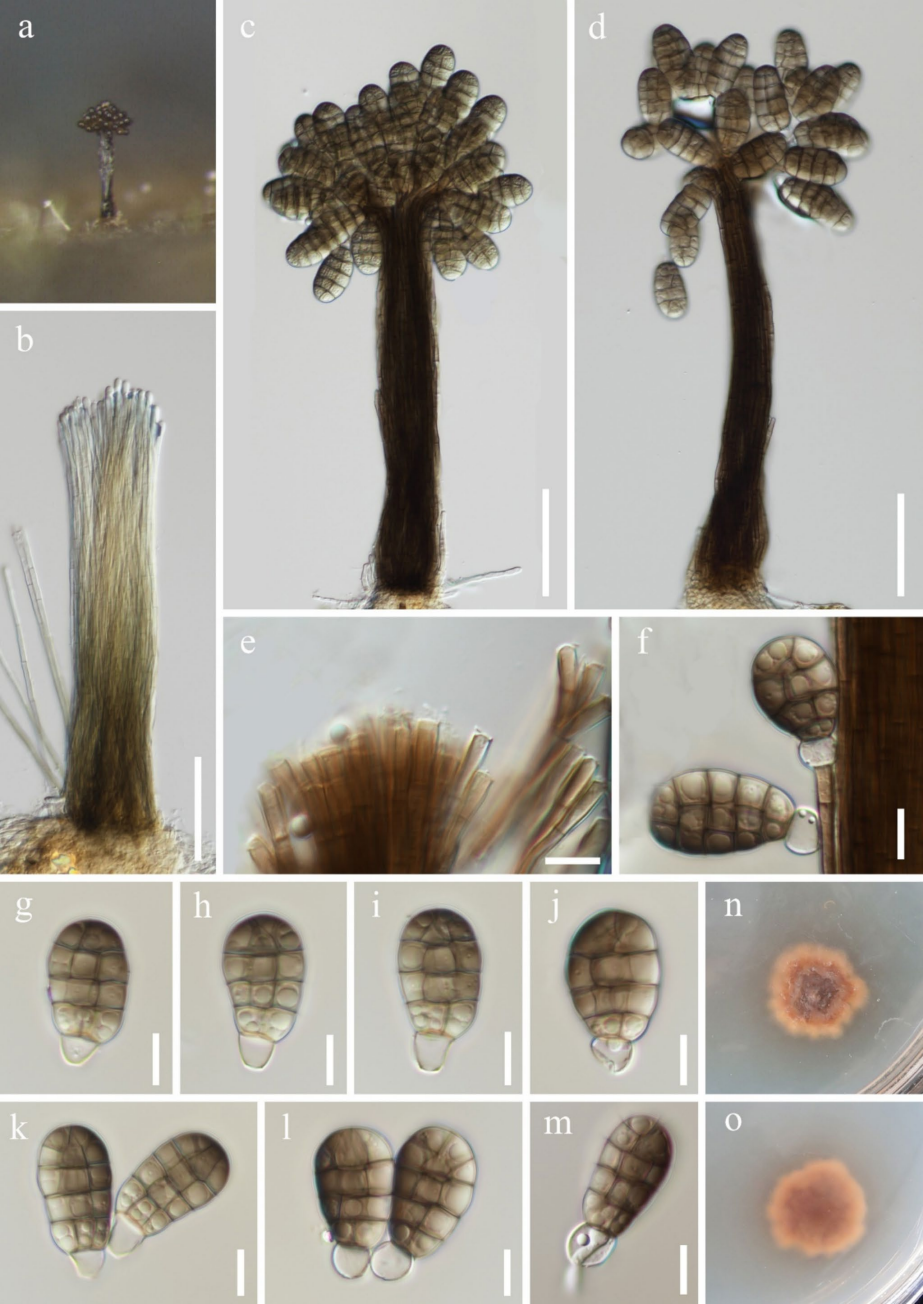
*Junewangia synnematica* (HKAS 144554, holotype). **(a)** Colony on the substratum; **(b)** Synnema; **(c,d)** Synnemata with conidia; **(e)** Conidiogenous cells; **(f)** Conidiogenous cell with conidia; **(g–l)** Conidia; **(m)** Germinating conidium; **(n)** Colony on the surface of PDA; **(o)** Colony on the reverse of PDA. Scale bars: **(b–d)** 50 μm; **(e–m)** 10 μm.

***Junewangia synnematica*** W.P. Wang & Z.L. Luo, sp. nov., Figure 8.

Fungal names number: FN572299.

Etymology: Referring to synnematous conidiophores of this fungus.

Holotype: HKAS 144554.

*Saprobic* on submerged decaying wood. **Asexual morph**: *Colonies* – erect, scattered, and brown to dark brown conidia gathered at the apex of synnemata. *Mycelium* - immersed, composed of aseptate, smooth, hyaline, unbranched hyphae. *Conidiophores* 102–137 × 2–2.3 μm ( $\bar{x}$ = 122.8 × 2.1 μm, *n* = 10), macronematous, synnematous, cylindrical, straight or slightly flexuous, multiseptate, unbranched, brown, slightly paler toward the apex, smooth and thick-walled. *Synnemata* 192–213 × 21–40 μm ( $\bar{x}$ = 201.5 × 29.7 μm, *n* = 5), erect, rigid, cylindrical, and dark brown. *Conidiogenous cells* 7.2–14.4 × 2.6–3.9 μm ( $\bar{x}$ = 11.3 × 3 μm, *n* = 10), monoblastic, integrated, determinate, terminal, cylindrical, brown. *Conidia* 21–28 × 13–17 μm ( $\bar{x}$ = 26.1 × 14.7 μm, *n* = 40), acrogenous, solitary, broadly ellipsoidal to pyriform, muriform, smooth-walled, with longitudinal and transverse separation; oblique septate at the apex row, slightly constricted at septum, composed of 3 columns and 4 (−5) rows of cells; broadly rounded apical, brown, guttulate; with a globose to subglobose, 6.3–7.7 μm in diameter, hyaline, and thin-walled separating cell connect to conidiogenous cells, becoming cuneiform base when mature. **Sexual morph**: Undetermined.

*Culture characteristics*: Conidia germinating on PDA within 24 h and germ tubes produced from the base. Colonies on PDA reaching 10 mm diameter after 6 weeks at room temperature. Colonies on the surface of PDA, irregular edges, umbellate, dry, reddish-brown to brown; smooth, reddish-brown to brown from reverse.

*Material examined:* China, Yunnan Province, Qujing City, Luoping County (25°01′52.57″N; 104°42′47.40″E), on unknown submerged decaying wood in a freshwater river, 15 July 2023, Fa-Li Li, S-5643 (HKAS 144554, holotype), ex-type culture, KUNCC 23-16608; Wenshan Zhuang and Miao Autonomous Prefecture, Malipo County (23°49′99.99″N; 104°97′74.49″E), on unknown submerged decaying wood in Dahe River, 28 February 2024, Ting-Xiang Liu, S-6470 (HKAS 144568, paratype), living culture, KUNCC 24-19062.

*Notes: Junewangia synnematica* is the first species in the genus *Junewangia* which has synnematous conidiophores (Table 2); it is distinguished from other species in this genus (Baker et al., 2002; Xia et al., 2017; Song et al., 2018a; Dong et al., 2021). In the phylogenetic analysis, *J. synnematica* (KUNCC 23-16608 and KUNCC 24-19062) clustered with *Dictyosporella hydei*, *J. aquatica*, *J. guangxiensis*, and *J. thailandica* in the same clade with 97% ML and 1.00 PP support (Figure 2). A comparison of the ITS sequence of the ex-type strain of *J. synnematica* with *J. aquatica* (HFJAU 0700) and *J. guangxiensis* (KUNCC 24-18376) (as *D. hydei* and *J. thailandica* lack ITS sequence in GenBank) showed differences of 6.73% (37/550 bp, nine gaps) and 4.93% (27/548 bp, seven gaps), respectively. We therefore introduced *J. synnematica* as a new species based on morphological characteristics and phylogenetic analysis.

***Junewangia thailandica*** W. Dong, H. Zhang & K.D. Hyde, Mycosphere 12(1): 53 (2021), Figure 9.

**Figure 9:**
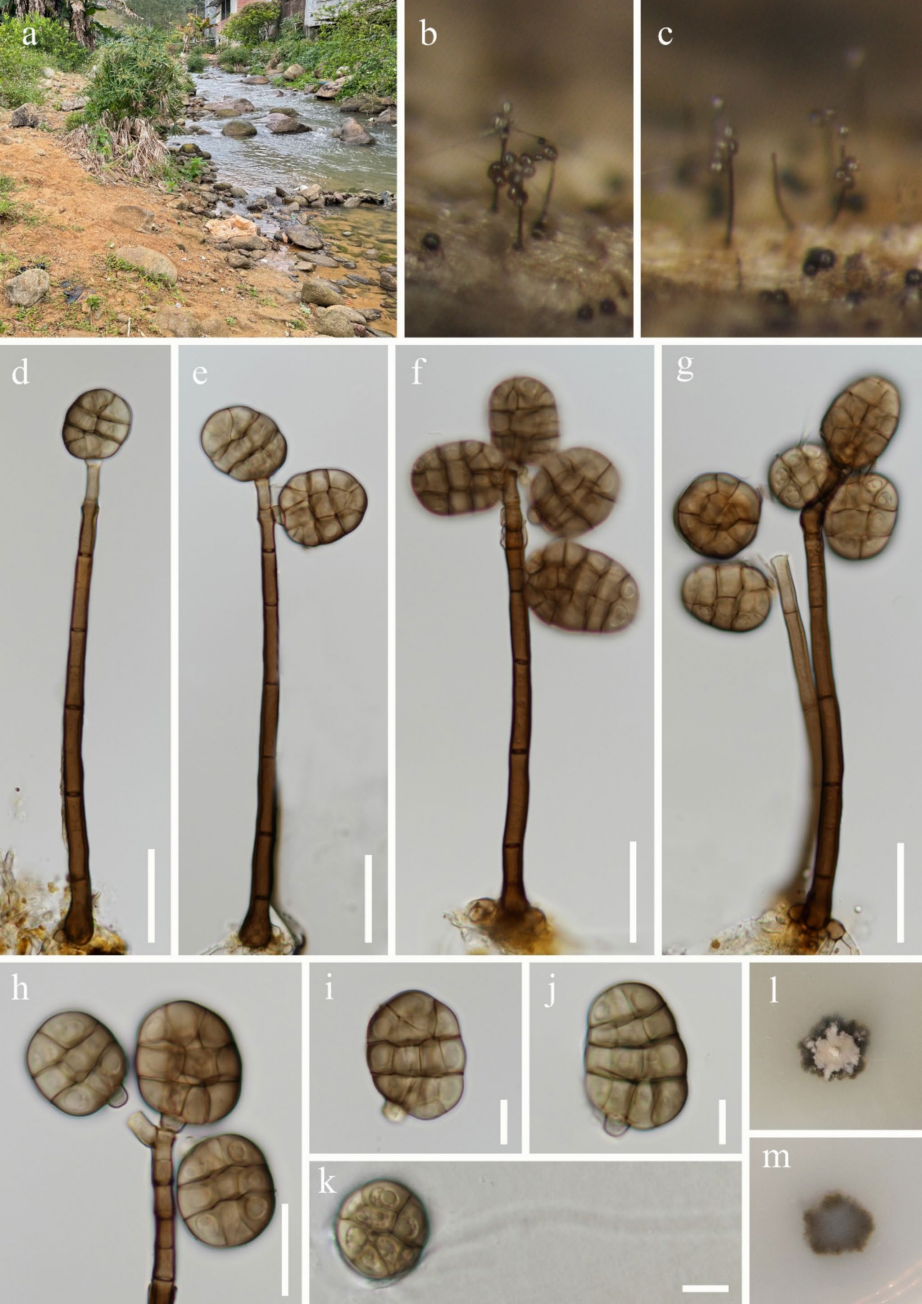
*Junewangia thailandica* (HKAS 144560). **(a)** Freshwater habitat; **(b,c)** Colonies on the substratum; **(d–g)** Conidiophores with conidia; **(h)** Conidiogenous cells with conidia; **(i,j)** Conidia; **(k)** Germinating conidium; **(l)** Colony on the surface of PDA; **(m)** Colony on the reverse of PDA. Scale bars: **(d–g)** 30 μm; **(h)** 20 μm; **(i–k)** 10 μm.

Fungal names number: FN558045.

*Saprobic* on submerged decaying wood. **Asexual morph**: *Colonies* - erect, scattered, brown, globose conidia at the apex of conidiophores. *Mycelium* – partly immersed, partly superficial, composed of septate, smooth, branched, brown hyphae. *Conidiophores* 112–177 × 4.7–6.3 μm ( $\bar{x}$ = 148.3 × 5.4 μm, *n* = 20), macronematous, mononematous, cylindrical, straight or slightly flexuous, 5–7-septate, unbranched, brown, paler toward the apex, smooth and thick-walled, with 2–6 percurrent proliferations. *Conidiogenous cells* – monoblastic, integrated, terminal, cylindrical, pale brown, smooth-walled. *Conidia* 24–37 × 18–28 μm ( $\bar{x}$ = 29.4 × 22.7 μm, *n* = 50), acrogenous, solitary, broadly ellipsoidal to subglobose, muriform, smooth-walled, longitudinal or oblique and transverse separation, constricted at septum, composed 3 (−4) rows cells, broadly rounded apical, brown, guttulate, with a cuneiform basal cell. **Sexual morph**: Undetermined.

*Culture characteristics:* Conidia germinating on PDA within 24 h and germ tubes produced from surface. Colonies on PDA reaching 8 mm diameter after 8 weeks at room temperature. Colonies semi-immersed in PDA, irregular edges, surface flat with a small protrusion in the center, dry, dark brown with a layer of gray hyphae covering the surface; dark brown from reverse.

*Material examined:* China, Guangxi Zhuang Autonomous Region, Yulin City (22°24′48.70″N; 109°70′42.37″E), on unknown submerged decaying wood in a freshwater stream, 25 February 2024, Wen-Peng Wang, S-6449 (HKAS 144560), living culture, KUNCC 24-18384.

*Notes:* Phylogenetic analysis showed that our new collection (KUNCC 24-18384) clustered with the holotype of *Junewangia thailandica* (MFLU 15-2682) with 100% ML and 1.00 PP support (Figure 2). Morphologically, our new collection fits well with the conception of *J. thailandica* in having macronematous, mononematous, cylindrical, and unbranched conidiophores with several percurrent proliferations and broadly ellipsoidal to subglobose, muriform conidia with similar size (24–37 × 18–28 vs. 22–32.5 × 16.5–23 μm) (Dong et al., 2021). We therefore identify our new collection as *J. thailandica*, a species first described by Dong et al. (2021) from a freshwater habitat in Thailand, and this is a new geographical record in China.

## Discussion

In this study, we introduced two new species of the genus *Junewangia* based on morphological characteristics and phylogenetic analysis. Phylogenetic analysis showed that the four genera, *Dictyosporella*, *Jennwenomyces*, *Junewangia*, and *Sporidesmiella* were chaotically clustered, especially *Dictyosporella* and *Junewangia* (Luo et al., 2019; Dong et al., 2021; Hyde et al., 2023; Figure 2). This finding suggests that species identification within *Junewangiaceae* cannot be solely based on the phylogenetic analysis. As shown in Figure 1, the primary difference between *Dictyosporella* and *Junewangia* lies in the conidiophores, whereas their muriform conidia are not significantly different (Xia et al., 2017; Song et al., 2018a,b; Dong et al., 2021). Some genera with closely phylogenetic relationships have similar conidia produced from different conidiophores, for example, *Dendryphion* and *Torula* (Su et al., 2016, 2018). Of course, some taxa exhibit highly variable conidiophores, even within the same genus or species, for example, *Phaeoiseria* and *Pleurotheciella* (Crous et al., 2015, 2017; Boonmee et al., 2021; Shi et al., 2021; Wang et al., 2024).

When discussing these issues, we cannot overlook the influence of factors, such as altitude, latitude, temperature, and host, on fungal morphology. Some studies have found that the host can influence the fungal morphology, particularly on conidiophores (Réblová et al., 2016; Jayawardena et al., 2022; Li et al., 2025), and this phenomenon is also observed in *Junewangia* (Xia et al., 2017). Understanding the host’s influence could be a way to resolve the taxonomic issue between *Dictyosporella* and *Junewangia*, but there are still too few relevant species for reference. In addition, studying the sexual morph is another potential approach to solving the taxonomic problem of *Junewangiaceae*. Currently, the sexual morph is only reported in *Dictyosporella* (Zhang et al., 2017; Dong et al., 2021).

## Data Availability

The datasets presented in this study can be found in online repositories. The names of the repository/repositories and accession number(s) can be found in the article/supplementary material.

## Footnotes

^1^https://nmdc.cn/fungalnames/, accessed on 2 January 2025.
